# Experiences and actions of part-time firefighters’ family members: a critical incident study

**DOI:** 10.1136/bmjopen-2024-086170

**Published:** 2024-09-05

**Authors:** Emelie Lantz, Bengt Nilsson, Carina Elmqvist, Bengt Fridlund, Anders Svensson

**Affiliations:** 1 Department of Health and Caring Science, Linnaeus University, Växjö, Sweden; 2 Research School for Doctoral Students within the Swedish Rescue Services, The Swedish Fire Research Foundation, Stockholm, Sweden; 3 West Blekinge Fire and Rescue Service, Karlshamn, Sweden; 4 Centre of Interprofessional Collaboration within Emergency care (CICE), Linnaeus University, Växjö, Sweden; 5 Department of Forestry and Wood Technology, Linnaeus University, Växjö, Sweden; 6 Agunnaryd Voluntary Fire Brigade, Ljungby, Sweden; 7 Department of Research and Development, Region Kronoberg, Vaxjo, Sweden

**Keywords:** work satisfaction, health services, family, health

## Abstract

**Abstract:**

**Objectives:**

The purpose of this study was to describe the experiences and actions of part-time firefighters’ family members in rural areas in Sweden.

**Design:**

The study had an inductive descriptive design and used the critical incident technique.

**Setting:**

Rural areas, primarily served by a part-time fire station, across Sweden.

**Participants:**

The study included 25 participants (21 females and 4 males) with experiences of being a family member of a part-time firefighter. Family members who themselves served as firefighters were excluded.

**Results:**

Being a part-time firefighter’s family member was described into three main areas of experiences: ‘affecting everyday life’, ‘dealing with uncertainty’ and ‘being in this together’. Actions taken were divided into two main areas: ‘pursuing adaptations’ and ‘alleviating difficulties’.

**Conclusions:**

Family members of part-time firefighters faced increased responsibility at home, managing personal inconvenience and frustration. They offered emotional support for the firefighter, however, expressing a need for guidance on handling firefighters’ emotions and mental health after call-outs. Despite their crucial role, they often felt unrecognised by the fire and rescue service. Nonetheless, they took pride in their firefighter’s contribution to the community and noted positive impacts on the family.

**STRENGTHS AND LIMITATIONS OF THIS STUDY**

- The inductive design employed in the study facilitated heightened comprehension of an under-researched area, contributing to the expansion of knowledge in the field.
- The need for clearly defined critical incidents and accurate recollection by the participants is one limitation.
- A potential limitation of employing the critical incident technique in this study is that it may have narrowed the scope to specific high-stress events.

## Introduction

 Balancing work as a non-career firefighter, for example, part-time or volunteer firefighter, with main employment and family obligations is challenging. This difficulty can lead to stress and dissatisfaction, which may ultimately result in firefighter resignations.^1^,^3^ Even though these firefighters are the backbone of fire and rescue services (FRSs), especially in rural areas, recruiting and retaining them is a well-known challenge.^4^,^6^ In Sweden, most firefighters are paid part-time firefighters (PTFs), usually serving on-call for 1 week per month. In contrast to other emergency personnel, PTFs are on-call from home, so the work-family interface is more pronounced. PTFs play an important role in public safety in rural areas; initiating an early response to emergencies, collaborating with the emergency medical services and by bringing valuable knowledge of the community they are part of.^7 8^ However, PTFs struggle to find a balance between work as a firefighter and other commitments such as family time.^9^ The family is an essential element in firefighter retention because they provide emotional support, help manage work-life balance and offer flexibility regarding the job’s demands.^9^ This support helps firefighters cope with stress and manage their responsibilities, contributing to their overall well-being and job satisfaction, which in turn improves retention. There is a scarcity of research examining the interaction between family dynamics and high-risk occupations,^10 11^ such as PTFs. However, a systematic review in this field, concludes that the mental health and well-being of spouses, partners and children of emergency responders is significantly affected by the demanding and stressful nature of emergency response work. Family members often experience heightened levels of stress, anxiety and mental health challenges due to the unpredictable and high-risk nature of their loved ones’ professions.^12^

The literature examining the relationship between work and family has traditionally focused on the impacts on organisations and the individual employee.^13 14^ Existing research has rarely focused on the effects that firefighters’ service has on family members.^15 16^ Work and family cannot be viewed as separate spheres but rather as highly inter-related. The boundaries between these spheres are thus flexible, permitting factors such as stress to overflow from one sphere to another. There have been debates about whether work-related stress is transmitted from one family member to other family members.^17 18^ The workplace culture, its demands and organisational support influence how firefighters’ family members perceive the efforts and rewards of the job in relation to the families’ needs and resources.^18^ Family members’ mutual dependence and the availability of social support are factors that increase the family’s capabilities for managing challenges.^15^ PTFs adjust their lives to fit with the service, which requires the support and commitment of the family.^9^ Family members of PTFs play a vital supporting role, yet their experiences and actions remain poorly understood. This knowledge gap hinders policy development by FRSs in addressing firefighter retention. The purpose of this study was to describe experiences and actions of PTFs’ family members in rural areas in Sweden.

## Methods

The study had an inductive descriptive design^19^ and used the critical incident technique (CIT) developed by Flanagan.^20^

### Critical incident technique

The CIT was used to understand the experiences of, and actions taken by, family members of PTFs in critical situations related to the firefighters’ work situation. This method was created to describe experiences and actions in different contexts.^21^ The CIT visualises both experiences and the subsequent actions taken in a well-defined, retrospective situation that affected a human activity, either positively or negatively.^21^ Instead of reflecting attitudes or opinions, the goal of CIT is to characterise participants’ experiences and their actions taken during critical situations.

### Settings and participants

Even if PTFs also serve in urban areas, they typically work in rural and sparsely populated regions, responding to emergencies in their local communities. In this study specifically, rural denotes an area primarily served by a part-time fire station, which means that the size and population of these areas vary. The inclusion criteria were being a family member of a PTF. The study strived to enrol participants with a variety of individual, family and work characteristics across Sweden, that is, a purposeful sampling, including a total of 25 participants (table 1). Family members who were also serving PTFs were excluded. A family member was defined as a person denoted by the PTF as someone important for their work situation in their everyday life.

**Table 1:** Individual, family and work characteristics of part-time firefighters’ family members

Characteristics	Numbers
Sex	
Female	21
Male	4
Age (years)	
20–30	2
31–45	14
>45	9
Relationship to firefighter	
Partner with no children at home	3
Partner with children at home	21
Parent of firefighter	1
Time of living with the firefighter (years)	
<5	6
5–15	15
>15	4
Work sector	
Private	8
Public	15
Student	1
Retired	1

### Data collection procedure

FRS managers, with a geographical spread in Sweden, were informed about the study. In turn, they notified PTFs in their organisation, either all of them or a selected few, instructing them to contact someone in the research team. In some cases, the research team obtained the PTFs’ contact information directly. The firefighters acted as ‘gatekeepers’, deciding whether they wanted to ask their family member to participate. After obtaining contact information for the family members, one person in the research team made contact, explaining the study both orally and in writing. The participants did not receive any questions beforehand.

The interview guide was pilot tested with three participants, transcribed and discussed among the authors, to evaluate the guide. These interviews were then included in the study due to richness in content. The interviews were conducted via zoom or telephone by the first author. The study’s purpose was explained at the start of the interview. The participants were urged to provide descriptions of past situations that they considered had an impact and in which they took action, meaning situations that stood out from the ordinary. The interview guide contained a main question followed by nine questions about each critical incident according to Fridlund *et al*^21^ (table 2). Furthermore, probing and checking were used to provide well-developed, rich and detailed answers and to promote dialogue, such as “could you elaborate on that” or “explain what you mean by that”. All the interviews ended with a summary of the described situations being presented to the family members, with the participant being offered the opportunity to add more information or descriptions. The interviews lasted from 21 to 49 min. The interviews were audio recorded and the transcribed material was pseudo-anonymised before the data analysis process began within the research group. The audio file and text were stored on two separate encrypted servers (one for back up). The information letter included written information on handling personal data in compliance with the EU’s General Data Protection Regulation. The interviews were verbatim transcribed by the first author. Two participants dropped out before the scheduled interviews.

**Table 2:** Description of the main question and the following nine interview questions in the critical incident technique according to Fridlund *et al*^21^

	Interview questions
**Main question**	Describe a situation* that had positive or negative effects for you in relation to the part-time firefighters’ work situation.
1	Where did the incident happen?
2	Can you give a detailed description of what happened?
3	Why is this critical incident significant to you?
4	What did you do in connection with the incident?
5	What was your mindset during the incident?
6	What were your thoughts during and after?
7	What were your feelings during and after?
8	What did you find the most demanding aspect of the incident?
9	What has this incident meant to you since?

* A situation in this context is congruent with a critical incident according to Fridlund *et al.*^21^Fridlund et al (2017) .

### Data analysis

The analysis was done manually by the authors. The first author began by reading the interview transcripts multiple times, extracting and condensing experiences and actions and assigning specific codes manually.^21^ The aggregation of codes into subcategories, categories and main areas was also done through a manual, systematic sorting and individual analysis process. The mean number of critical incidents per participant were 4, generating 354 experiences and 151 actions. The experiences and actions were sorted and analysed separately, but the analysis process was the same for both. Codes relating to each other in terms of content were grouped together as subcategories at a descriptive level. Then similar subcategories were grouped together in categories. These categories were then grouped into main areas. The analysis of experiences included 32 subcategories, 7 categories and 3 main areas, while the equivalents for the corresponding actions were 13, 6 and 2, respectively. The authors conducted the analysis process together and discussed the results at every step of the analysis. The authors had thorough experiences of the CIT and was an interdisciplinary group. All authors were involved in the analysis process. It was a collaborative effort to scrutinising and systematising the data to validate the results at each step. All authors reached a consensus on the categories and areas that emerged, ensuring that the analysis was well-supported by the data. When a challenge or a diverse view arose, the authors revisited the transcribed interview to gain a comprehensive understanding and choose the most reasonable codes that best matched the data.

### Patient and public involvement

Patients or public were not involved in the design of this study. However, all participants were informed in the information letter that the results should be disseminated in scientific journals, at conferences and as popular science publications. All participants were given the opportunity to sign up to receive the future published material.

## Findings

Being a family member to a PTF was described according to experiences in three main areas: ‘affecting everyday life’, ‘dealing with uncertainty’ and ‘being in this together’ (table 3). The actions taken were in two main areas: ‘pursuing adaptations’ and ‘alleviating difficulties’ (table 4).

**Table 3:** An overview of main areas, categories and subcategories (with number of experiences) regarding experiences of part-time firefighters’ family members

Main area	Category	Subcategory (n)
Affecting everyday life	Increased burden in everyday life	Feeling demands in everyday life (7)
		Having increased responsibility for children (9)
		Accepting the situation (26)
		Needing extra support (8)
	Sense of restriction	Being in stand-by (3)
		Feeling limited (24)
		Being restricted by on-call time (10)
Dealing with uncertainty	Need for information	Wanting knowledge about how to provide support (7)
		Wanting information about the call-out (12)
		Relating to call-out confidentiality (10)
	Consequences of call-outs	Feelings of unpredictability (15)
		Relating to inconvenient call-outs (12)
		Worry during night-time call-outs (9)
		Being left alone (21)
		Needing supportive conversation afterwards (8)
Being in this together	Feeling negative emotions	Feeling frustration (10)
		Feeling disappointment (11)
		Feeling worry (25)
		Feeling excluded (5)
		Experiencing stress (10)
		Feeling relationship friction (7)
	Shared family concerns	Needing a family collaboration (10)
		Seeing the firefighters’ joy (12)
		Relating to financial aspects (3)
		Wanting a give and take perspective (5)
		Enabling the work situation (8)
		Being in a community for families (16)
		Wanting mutual understanding (4)
	New perspective on life	Relating to the community’s needs (16)
		Feeling pride (12)
		Seeing positive effects (6)
		Receiving a deeper perspective on life (13)

**Table 4:** An overview of main areas, categories and subcategories (with number of actions) regarding actions taken by part-time firefighters’ family members

Main area	Category	Subcategories (n)
Pursuing adaptions	Navigating increased obligations	Taking responsibility at home (16)
		Taking responsibility for children (21)
		Seeking external support (4)
	Organising solutions	Finding alternative solutions (5)
		Coping alone (4)
	Planning ahead	Arranging plans and structure (23)
		Seeking information about the call-out (7)
	Adjusting to the situation	Setting aside main work (9)
		Adjusting in everyday life (16)
Alleviating difficulties	Communicating to resolve friction	Seeking a communicative approach (9)
		Having conversations after call-outs (19)
	Supporting the firefighter	Being there (11)
		Assisting during call-outs (7)

### Experiences being a family member of a PTF

#### Affecting everyday life

Being a family member of a PTF affected everyday life. It involved navigating daily demands and limitations during the on-call periods. This was particularly true when younger children were involved, as the firefighter could not care for them alone at home due to potential call-outs that required an immediate response. Consequently, the family member had to stay physically close to home or arrange backup support in case of call-outs, necessitating careful planning and adjustments:

Now it was my responsibility entirely, and he couldn’t do anything with the children.

This was experienced as an everyday problem that increased challenges and limitations, leading to the family member feeling restricted or being in a state of stand-by. However, there was an acceptance of this burden. It became a part of everyday life, even if it was inconvenient:

Life was like this all the time while having small children. It was a part of everyday life.

Balancing these aspects created a daily puzzle that demanded effort and imposed constraints on the family member, resulting in a sense of reduced ability to be spontaneous. Family members felt the need to align their lives with the on-call time. Although accepting it as a way of life, family members felt increased responsibility and required extra support to make it work.

#### Dealing with uncertainty

Family members of PTFs dealt with uncertainty due to call-outs. Firefighters encountered challenging situations and emotions during emergencies, and these difficult feelings extended into their home life, affecting family members. Coping with the mental health aspects of these experiences was challenging for family members, who often lacked the relevant knowledge or external support. However, being a support for the firefighter by engaging in open communication about their feelings became part of the family members’ experiences. Family members found themselves left alone whenever the firefighter was called out. These call-outs could occur at highly inconvenient times, leading to stress or frustration for family members. The family members had no information about how long the firefighter would be gone, ranging from minutes to a whole day. In an attempt to cope with the situation, family members wanted information about the call-out. Due to confidentiality, they were frequently left with uncertainty and gaps in knowledge:

I wanted to get a context, but he couldn’t provide it.

Night-time call-outs further impacted family members, disrupting their sleep:

I found it very uncomfortable when the alarm went off at night. I thought it was terribly unpleasant because then I had nothing to distract my thoughts, and they usually spun out of control.

#### Being in this together

Being a family member of a PTF involved being in it together. When someone took on the role of a PTF, it became a family decision that aimed to provide the necessary support during on-call periods so that everyday life still functioned. The increased workload on other family members led to negative emotions, such as frustration or disappointment:

But the emotions linked to it were, well, disappointment. That it didn’t turn out the way one had imagined.

It also involved stress and worry about the firefighter, both due to call-outs and long-term health consequences. Family members did not participate in the firefighter’s work context, leading to feelings of exclusion:

It was obvious, it wasn’t strange, but you felt left out. Because they had something together, and they’d experienced things together that no one else could come close to.

The on-call periods created friction in the relationship and everyday life, leading to a multifaceted experience of stress for the family members.

Nevertheless, it was a shared matter for the family, a joint decision. There was a financial aspect to the work, and a sense of give and take was involved. Firefighters often conveyed a genuine sense of joy in their work, which tended to positively influence the family. Family members described witnessing the firefighters’ joy and had a desire to make everyday life work so they could continue. Becoming and continuing to work as a PTF was often a mutual decision, highlighting the importance of communication and consensus within the family:

I thought it was a family thing to be part-time firefighters, you know. I thought it was, well, I mean a collective sacrifice.

The firefighters’ work situation also led to family members meeting new people, creating a new community context for firefighter families. Additionally, although family members felt negative emotions due to the firefighters’ work situation, it also brought positive effects and another perspective on life. PTFs that handled life-and-death situations brought home a new perspective on life and provided food for thought, influencing the family in a positive manner. Being a family member of a PTF was emotionally taxing, as family members navigated a delicate balance between their own inconveniences in everyday life and the community’s need for safety. However, family members expressed a sense of pride that also influenced their positive mindset.

### Actions taken due to being a PTF’s family member

#### Pursuing adaptations

Being a family member of a PTF entailed a pursuing way to adapt everyday life. These include taking increased responsibility for the home, children and the organisation of daily life. Due to the firefighter’s commitment to a specific proximity to the fire station, the family member became primarily responsible for tasks such as children’s dentist appointments, grocery shopping or walking the dog. This impacted both family time at home and their work commitments, requiring early departures for child pickups or facilitating transportation to activities. In addition to the role of the main caregiver for the children during on-call periods, the family member also took on the responsibility of addressing the children’s potential emotions, thoughts and disappointments when the firefighter had to respond to a call-out. During these situations, the family member had to handle matters independently, cultivating a problem-solving mindset even in the face of discouragement. Proactive planning before on-call periods became essential to effectively manage these heightened responsibilities. To be able to plan ahead when the firefighters left for a call-out, the family members sought information about the call-out and, if necessary, explored external support options:

One became quite skilled at planning and organising, you know, to make it work.

Family members set aside other commitments in favour of the firefighters on-call time, even adapting their work:

After all, his on-call service did lead to me changing my job as well.

Regardless of whether the family had children at home or not, the PTF’s family often did things separately due to the firefighter’s geographical limits around the fire station. Family members put their life and activities on hold during on-call periods.

#### Alleviating difficulties

Family members of PTFs alleviated the difficulties of family life. The family member was a significant support to the PTF, both in everyday life and emotionally. There was often a need for supportive communication after a challenging call-out:

So it was noticeable when you lived together. Something had happened, you know. And then you didn’t really know where the boundaries were, how much could you ask?

Family members did not have knowledge about what to do and where to turn if they noticed the firefighter having difficult emotions and mental health challenges after a call-out. They wanted to have a supportive conversation without impacting the confidentiality of the call-out. Family members also took action to assist during call-outs, providing food on the go or calling the main employer to inform them about the firefighter being away or late. Communication was vital to resolve friction in relationships due to the firefighter’s work situation. Even starting to work as a PTF was a joint decision, there were also ongoing conversations about whether he or she should continue as a firefighter due to the inconvenience.

## Discussion

The study findings shed light on the multifaceted role played by family members of PTFs by describing their experiences and actions. The findings reveal five main areas that offer valuable insight into this under-researched group, with both theoretical and practical implications.

### Study findings

The findings highlight the complex interplay of responsibility, support and communication among family members of PTFs, which is discussed in the sections below.

The study findings underscore that being a PTF’s family member involves a significant increase in responsibility at home. The family member often becomes the primary caretaker for children and takes charge of daily tasks. The study reveals the need for proactive planning and organisation by family members to effectively manage the increased responsibilities of on-call periods. This corresponds with other literature that suggests that family members made sacrifices, and they shared strategies they use to mitigate the impact of their firefighter’s work.^22^ One approach involved anticipating unexpected disruptions to their shared family and social time; a conscious decision to plan flexibly and actively preparing for unforeseen events and intentionally scheduling downtime when their firefighter was on-call.^22^ The day-to-day pressures of emergency responder occupations significantly impact the health and well-being of spouses/partners and children, with spillover of work stress into family life, affecting mental health, well-being and family functioning.^12^ Spouses often bear the burden of household responsibilities alone due to irregular work schedules.^23^ However, this study also highlights the increased responsibility and the inconvenience that may be entailed in actioning this planning. Even if the family member is prepared for the firefighter leaving at any time, it nevertheless involves frustration or disappointment. To address some of this frustration and to alleviate the family member’s need for planning, a smart phone application with a time prognosis is valuable. Several Swedish FRSs use an application called *Respons*, designed to enhance communication and information management for emergency services.^24^ Granting family members limited access to this tool could be beneficial, as it would aid in planning and reduce uncertainty through improved forecasting.

Family members emerge as a crucial source of emotional support for PTFs. The study reveals that after challenging call-outs there is a noticeable need for supportive communication. However, there is a recognition of the delicate balance between offering support and respecting the confidentiality of the call-out. These findings correspond with other literature in the work-family interface. Family-provided social support plays a crucial role in preserving the health and well-being of emergency responders.^12^ While support may come from various sources, it appears that firefighters particularly value support from family and significant others, with support from co-workers and the organisation following closely behind.^25^

Firefighters face significant psychological and physical challenges, and they must deal with irregular work schedules, time constraints and the need to perform in emergency situations regardless of the staff and resources that are available.^11^ Furthermore, there were details about how emergency service workers experienced a sense of disengagement and emotional distance from their family members on returning from work.^26^ Stress and symptoms experienced by the firefighter from a traumatic event can lead to a trauma response by family members; firefighter symptoms like mood swings and irritability are distressing enough to elicit a certain degree of traumatic reaction from their family members.^27 28^ However, this study adds to the complexity of highlighting the need for information and knowledge for the family member in order to be able to address firefighters’ mental health and difficult emotions that arise from call-outs. The family is impacted by firefighters’ behaviour and moods, expressing the need for communication and alleviating emotions. Even though the literature highlights the importance of family members’ support for the firefighter’s well-being,^22 26^ there is also a constant tension for the family members. They must balance their personal inconveniences in daily life with the community’s need for safety delivered by the PTFs. This creates a conflict between the safety of others and the individual’s own needs.

Family members experience inconveniences and increased responsibility at home, but it is hard to argue against the important work of PTFs in the community. This contrasts with families of full-time firefighters, who follow a regular schedule of being at work and then returning home during their off-time. PTFs, on the other hand, maintain regular work schedules and additionally have on-call responsibilities from home. Therefore, recognising and valuing the contributions of family members to PTFs are vital. Maintaining a healthy work-family balance is crucial for the long-term retention of firefighters, as it reduces the strain on their personal lives and encourages continued participation.^3^ Furthermore, FRSs should strive to reduce the inconvenience for family members by offering support, recognition and practical initiatives, such as flexible schedules. By establishing support systems for family members, the FRS can help them to cope with the emotional challenges they face, for example, counselling services or resources for managing stress and anxiety.

This study describes how family members of PTFs see the joy their work brings which, in turn, has spill-over effects on the family. Family members also feel a sense of pride and contribution to the community. This corresponds with other literature that highlights how family members show strong support for and pride in the firefighters’ work.^18^ Experiencing job satisfaction and a positive work environment also fosters a constructive atmosphere at home, resulting in the enjoyment of quality family time.^29 30^ There is a mutual connection between family members and the FRS.^22^ Family members employed terms like ‘our’, ‘we’ and ‘all of us’, when discussing the firefighters’ organisation, and exhibited a thorough understanding of factors such as community priorities, policies, operational procedures and specialised terminology. However, this does contrast with the findings were family members instead described feelings of being a bit set aside, or excluded or not appreciated by the FRS. They also felt that PTFs were strict about confidentiality, often excluding information when communicating with family members after a call-out. This draws parallels to a study on military spouses and families, who may also lack detailed information about the specifics of the employment due to security restrictions. This lack of information can significantly impact communication within the family unit.^31^ The quality of communication in relationships involving emergency responders is significantly linked to their emotional responses and overall well-being. When both parties perceive a secure attachment, it leads to enhanced constructive communication and greater marital satisfaction.^23^ To address these challenges, FRSs need to consider organising workshops or support groups that allows families to share their experiences and strategies, fostering new networks and relationships and helping them communicate about their feelings. Furthermore, another possibility is to implement more flexible on-call schedules to better accommodate the daily challenges faced by family members. In addition, the introduction of a carpooling system is a fitting help for families with their everyday activities.

### Study methods

The study’s trustworthiness was achieved by addressing the concepts of credibility, dependability, confirmability and transferability throughout the research process.^32^

To strengthen the credibility of the study, interviews were chosen as the method for data collection, as it allowed participants to express their thoughts in detail, facilitated by follow-up questions. Saturation was not addressed in the manner of Grounded theory. Instead, the number of critical incidents was determined by the study’s purpose, that is a purposeful sampling, rather than focusing on the quantity of participants, in accordance with Flanagan’s approach.^20^ A potential limitation of employing the CIT in this study is that it may have narrowed the scope to specific high-stress events experienced by PFTs, potentially overlooking the broader impact of their work on daily life for both PTFs and their families. To strengthen the study’s dependability, participants from different parts of Sweden were intentionally chosen to represent a diverse range of age groups and years as a PTF’s family member. However, accurately and completely recollecting significant situations poses a challenge. There is a risk that the vividness and clarity of these memories may diminish over time, affecting the dependability of the data. The confirmability was reinforced by the authors performing the analysis together. The description of outcomes was discussed with all the authors to address biases and contemplate perspectives. The number of critical incidents gathered is determined by the scope of the study rather than the quantity of participants involved.^20^ The findings are deemed potentially transferable to other contexts involving non-career firefighters, such as volunteer firefighters. Although non-career firefighters do not usually serve on-call, their families are still impacted by the uncertainty surrounding call-outs, which creates a parallel to the experiences of PTF families.

## Conclusion

This study underscores the profound impact on the daily lives, emotional well-being and family dynamics of PTFs’ families. Family members balance their personal inconveniences with the community’s safety needs, navigating the conflict between their own needs and the demands of the firefighter’s role. Despite these challenges, they strive to provide emotional support and maintain open communication to address mental health aspects. The decision to become a PTF is a collective family commitment, fostering mutual support and shared responsibility.

Further research is needed regarding the work-life balance and identifying ways to improve it for PTFs and their families. Longitudinal studies will improve the understanding of the long-term effects of having a family member who works as a firefighter, including psychological well-being, equality, family dynamics and overall quality of life. Also, future research should further examine the effectiveness of support programmes designed specifically for the families of PTFs. This ought to include interventions to address the heightened responsibility at home, strategies for managing inconveniences and methods for enhancing support. Future research could benefit from incorporating a more comprehensive exploration of the holistic experiences of PFTs and their families, outside critical incidents, to provide a more nuanced understanding of the impacts of firefighting careers.

## Data Availability

No data are available.
