# Supplementary material for: Experiences and actions of part-time firefighters’ family members: a critical incident study
Source: BMJ Open. 2024 Sep 5;14(9):e086170. doi: 10.1136/bmjopen-2024-086170 (PMC11381642; doi:10.1136/bmjopen-2024-086170)
Supplement: Supplementary file 1 [file bmjopen-2024-086170.reviewer_comments.pdf]

PEER REVIEW HISTORY

BMJ Open publishes all reviews undertaken for accepted manuscripts. Reviewers are asked to complete a checklist review form and are provided with free text boxes to elaborate on their assessment. These free text comments are reproduced below.

ARTICLE DETAILS

**TITLE (PROVISIONAL)**	Experiences and actions of part-time firefighters’ family members: a critical incident study
**AUTHORS**	Lantz, Emelie; Nilsson, Bengt; Elmqvist, Carina; Fridlund, Bengt; Svensson, Anders

VERSION 1 - REVIEW

**REVIEWER NAME**	Sharp, Marie-Louise
**REVIEWER AFFILIATION**	King's College London, Psychological Medicine
**REVIEWER CONFLICT OF INTEREST**	None
**DATE REVIEW RETURNED**	19-Jun-2024

**GENERAL COMMENTS**	Thank you for your work assessing the experiences and actions of part-time firefighter family members. I think this is a very neat study that provides additional data on a population group where there is little evidence currently. Whilst there may be more evidence regarding firefighters and emergency responder families more broadly - the difference here is that this group are part-time and I believe that has important implications.<br><br>I provide some further notes below as to how I believe this piece could be strengthened for publication.<br><br>Intro<br>- A statement is made line 21 that family is an essential element in firefighter retention but would be helpful to have a second part of the sentence explaining why as readers are left to guess why this might be.<br>- Noted scarcity of research examining family dynamics and high risk occupations – I think this is true in part, but note that our recent systematic review is not cited or integrated in this piece of work which addresses some of this topic - Sharp ML, Solomon N, Harrison V, Gribble R, Cramm H, Pike G, Fear NT. The mental health and wellbeing of spouses, partners and children of emergency responders: A systematic review. PLoS one. 2022 Jun 15;17(6):e0269659.<br>- Additionally our report that covers work/family conflict in emergency responder populations – which provides a systematic review - Sharp ML, Harrison V, Solomon N, Fear N, King H, Pike G. Assessing the mental health and wellbeing of the emergency responder community in the UK. I believe your report could benefit from possibly reading Chapter 7.

	- Finally – there is a lot of work that assesses family dynamics in Armed Forces occupations (i.e. a high risk occupation) – hence you may need to be more specific about the occupations you mean - but there is a lot to learn from the experience of Armed Forces Reserve families and Armed Forces families more generally - so I would be interested if you might include some of this in this work for comparison. This article gives some background on military families but there is a large literature aside from this - Gribble R, Connelly V, Fear NT. Living a life less ordinary: What can the families of essential workers responding to COVID-19 learn from UK military families?. Journal of Military, Veteran and Family Health. 2020 Nov 1;6(S2):27-34.<br><br>Method<br>- Please add a citation for inductive descriptive design or a more specific analysis process for example reflexive thematic analysis as currently it is not clear.<br><br>Data collection procedure<br>- How were the research team in some cases able to contact PTF's directly? Were their contacts available publicly? I ask in terms of data protection requirements of participant contact.<br>- Is there a typo – should it be 'contact someone in the research team' ?<br><br>Data analysis<br>- Can you specifiy which researchers conducted the coding analysis process<br><br>Discussion<br>- Page 9 – I enjoy the paragraph discussing the family as crucial support for PTF's -the situation regarding confidentiality and the tension this brings regarding communication which is key to providing support – I believe there are similarities of this with Armed Forces families where sometimes the family member cannot know about the specifics of deployment which can hamper communication and support offered. Ormeno MD, Roh Y, Heller M, Shields E, Flores-Carrera A, Greve M, Hagan J, Kostrubala A, Onasanya N. Special concerns in military families. Current psychiatry reports. 2020 Dec;22:1-7.<br>- Additionally our review collated a whole section on communication between emergency responder couples which I believe would be helpful for this paragraph - Sharp ML, Solomon N, Harrison V, Gribble R, Cramm H, Pike G, Fear NT. The mental health and wellbeing of spouses, partners and children of emergency responders: A systematic review. PLoS one. 2022 Jun 15;17(6):e0269659.<br>- Sense of pride in the work is a common theme in these roles – also identified as a theme in our review above.<br>- I would like to see the discussion draw out more the similarities and differences of part-time compared to full-time firefighter families for example - as I think the part-time element and rurality is key - some Australian part-time firefighter literature may help speak to this.<br><br>Discussion of methods<br>- I think the authors could add a limitation that by focusing on critical incidents, they may have missed out on data that addresses more of the day to day life of PFT's and their families where no doubt their

	work as a firefighter still has impact more broadly than just when they are out on a call.<br>- Lastly I am curious that discussion of impact on children did not seem to arise in interviews hugely – could the authors note this and perhaps comment why this might be?<br><br>Thank you again - I enjoyed reading the work and wish you well with it.

**REVIEWER NAME**	Jones, Sara
**REVIEWER AFFILIATION**	University of Arkansas for Medical Sciences, College of Nursing
**REVIEWER CONFLICT OF INTEREST**	None.
**DATE REVIEW RETURNED**	02-Jul-2024

**GENERAL COMMENTS**	Well written and very important work. Various concerns, should be easy to address.<br><br>**Data Collection Procedure** (page 5): Looking for more details about the interviews. Were they recorded? If recorded, both audio and video (Zoom)? How were recordings ethical compliant and saved? How were interviews transcribed? Reviewed for accuracy? These are all important things to consider for rigor and validity of qualitative data. Why did you stop at 25? Saturation not addressed.<br><br>Regarding the interview guide: Who developed it? Was it reviewed? Pilot tested? Suggest adding the main question as presented in the text to the table as the main probe.<br><br>**Data Analysis** (pages 5-6): How was analysis done? Was it by hand or using a qualitative analysis software? How many researchers completed the coding? How was inter-rater reliability determined? (Again, important to demonstrate reliability of qualitative methods.)<br><br>**Tables 3 & 4**: Suggest moving it to FINDINGS sections, after first short paragraph.<br><br>**Findings** (pages 6-8): This is more so regarding Tables 3 & 4. First, I'd suggest reorganizing the columns in the table. "Main Area" on the left; also recommend calling this a "Main theme." Then Category, and sub-category on right. I would also recommend emphasizing the raw data (direct quotes) more. Within the text, they don't really stand out. Consider making them a separate line, or making a 4th column to the right in the table with the example quotes.<br><br>**Discussion and Discussion of Findings** (pages 8-9): Don't just repeat the results. This section needs more depth. You mention how the findings "highlight" this and that, but how? Be specific. What is the "so, what?" of the findings.<br><br>**Discussion of methods** (pages 9-10): The very first sentence (lines 51-52) left me hanging. Suggest adding paragraph (page 10, lines 14-30) immediately following here. Then address each one of those

	constructs, in order, with the specifics. Page 10, line 21: should be "the majority of PTFs WERE men."<br><br>**Conclusions and Implications** (pages 10-11): The first paragraph is just repeat of the results again. Instead, synthesize the review of literature and study results collectively.<br><br>The next sections (page 10, line 44, to page 11, line 6) is what I would have liked to see in the Discussion section. Consider putting it there and expanding on the recommendations based on the evidence and previous literature.<br><br>**COREQ Checklist** does not appear correct. Unable to find Item #1 on page 11. Item #2: Credentials not listed on title page. This is PhD, MD, etc. Item #5: cannot locate training and experience. This could be added where describing who conducted the interview and analyzed the data, if needed. #10: Sampling method not identified. #13: not addressed. #17 and #19: not addressed. Also see my comments above regarding data collection. #20: Why are field notes during interviews "N/A"? Field notes are important in qualitative studies. #22: Saturation not addressed. #24: not addressed.<br><br>Editorially: Be sure to review for punctuation in right locations (periods and commas inside quotation marks). Consider use of oxford comma.

VERSION 1 – AUTHOR RESPONSE

**Reviewer 1 comments**	**Reply**
**Intro**	
A statement is made line 21 that family is an essential element in firefighter retention but would be helpful to have a second part of the sentence explaining why as readers are left to guess why this might be.	Thank you for your valuable comment. This is rewritten as follows: The family is an essential element in firefighter retention because they provide emotional support, help manage work-life balance, and offer flexibility regarding the job's demands [9]. This support helps firefighters cope with stress and manage their responsibilities, contributing to their overall well-being and job satisfaction, which in turn improves retention.
Noted scarcity of research examining family dynamics and high risk occupations – I think this is true in part, but note that our recent systematic review is not cited or integrated in this piece of work which addresses some of this topic - Sharp ML, Solomon N, Harrison V, Gribble R, Cramm H, Pike G, Fear NT. The mental health and wellbeing of spouses,	Thank you for addressing this valuable review. This is added as a reference in the text.

partners and children of emergency responders: A systematic review. PLoS one. 2022 Jun 15;17(6):e0269659.	
Additionally our report that covers work/family conflict in emergency responder populations – which provides a systematic review - Sharp ML, Harrison V, Solomon N, Fear N, King H, Pike G. Assessing the mental health and wellbeing of the emergency responder community in the UK. I believe your report could benefit from possibly reading Chapter 7.	Thank you for your valuable input on this literature. This reference is also added in the discussion section.
Finally – there is a lot of work that assesses family dynamics in Armed Forces occupations (i.e. a high risk occupation) – hence you may need to be more specific about the occupations you mean - but there is a lot to learn from the experience of Armed Forces Reserve families and Armed Forces families more generally - so I would be interested if you might include some of this in this work for comparison. This article gives some background on military families but there is a large literature aside from this - Gribble R, Connelly V, Fear NT. Living a life less ordinary: What can the families of essential workers responding to COVID-19 learn from UK military families?. Journal of Military, Veteran and Family Health. 2020 Nov 1;6(S2):27-34.	Thank you for your valuable input on this literature. While we appreciate good advice, in this case related for military families, we have chosen not to explore this further due to word limitations in this article.
**Method**	
Please add a citation for inductive descriptive design or a more specific analysis process for example reflexive thematic analysis as currently it is not clear.	Polit and Beck (2020).<br><br>This reference is added.
**Data collection**	
How were the research team in some cases able to contact PTF's directly? Were their contacts available publicly? I ask in terms of data protection requirements of participant contact.	As stated in the data collection section, in some cases, the research team obtained the PTFs' contact information directly from the Fire and Rescue Service managers. This contact information was handled confidently as stated in the ethical consideration section.
Is there a typo – should it be 'contact someone in the research team' ?	Thank you, this is corrected.
**Data analysis**	
Can you specifiy which researchers conducted the coding analysis process	The first author conducted the coding which is now clarified in the text.

**Discussion**	
Page 9 – I enjoy the paragraph discussing the family as crucial support for PTF's -the situation regarding confidentiality and the tension this brings regarding communication which is key to providing support – I believe there are similarities of this with Armed Forces families where sometimes the family member cannot know about the specifics of deployment which can hamper communication and support offered. Ormeno MD, Roh Y, Heller M, Shields E, Flores-Carrera A, Greve M, Hagan J, Kostrubala A, Onasanya N. Special concerns in military families. Current psychiatry reports. 2020 Dec;22:1-7.	This is added in the discussion section.<br><br>This draws parallels to a study about military spouses and families, whom also may not be informed about the specifics of the employment due to security restrictions. This lack of information can significantly impact communication within the family unit.
Additionally our review collated a whole section on communication between emergency responder couples which I believe would be helpful for this paragraph - Sharp ML, Solomon N, Harrison V, Gribble R, Cramm H, Pike G, Fear NT. The mental health and wellbeing of spouses, partners and children of emergency responders: A systematic review. PLoS one. 2022 Jun 15;17(6):e0269659.	This is added in the discussion section.<br><br>The quality of communication in relationships involving emergency responders is significantly linked to their emotional responses and overall wellbeing. When both parties perceive a secure attachment, it leads to enhanced constructive communication and greater marital satisfaction.
Sense of pride in the work is a common theme in these roles – also identified as a theme in our review above.	Thank you for the comment. Pride is discussed in the section.
I would like to see the discussion draw out more the similarities and differences of part-time compared to full-time firefighter families for example - as I think the part-time element and rurality is key - some Australian part-time firefighter literature may help speak to this.	Thank you for the valuable input. This is added and addressed in the discussion.<br><br>This contrasts with families of full-time firefighters, who follow a regular schedule of being at work and then returning home during their off-time. Part-time firefighters, on the other hand, maintain regular work schedules and additionally have on-call responsibilities from home.
**Discussion of method**	
I think the authors could add a limitation that by focusing on critical incidents, they may have missed out on data that addresses more of the day to day life of PFT's and their families where no doubt their work as a firefighter still has impact more broadly than just when they are out on a call.	This is added in the method discussion.<br><br>A potential limitation of employing the CIT in this study is that it may have narrowed the scope to specific high-stress events experienced PFTs, potentially overlooking the broader impact of

	their work on daily life for both PTFs and their families. Future research could benefit from incorporating a more comprehensive exploration of the holistic experiences of PFTs and their families to provide a more nuanced understanding of the impacts of firefighting careers.
Lastly I am curious that discussion of impact on children did not seem to arise in interviews hugely – could the authors note this and perhaps comment why this might be?	The children's perspective did come up during the interviews in terms of managing their disappointments and emotions. However, it was not a primary focus in the interviews, mainly because the discussions were centred around the perspectives of the adult participants.
**Reviewer 2 comments**	**Reply**
**Data Collection Procedure**	
Looking for more details about the interviews. Were they recorded? If recorded, both audio and video (Zoom)? How were recordings ethical compliant and saved? How were interviews transcribed? Reviewed for accuracy? These are all important things to consider for rigor and validity of qualitative data.<br><br>Why did you stop at 25? Saturation not addressed.<br><br>Regarding the interview guide: Who developed it? Was it reviewed? Pilot tested? Suggest adding the main question as presented in the text to the table as the main probe.	This is added under data collection:<br><br>The interviews were audio recorded and the transcribed material was pseudo-anonymized before the data analysis process began within the research group. The audio file and text were stored on two separate encrypted servers (one for back up). The information letter included written information on handling personal data in compliance with the EU's General Data Protection Regulation (GDPR). The interviews were verbatim transcribed by the first author.<br><br>Saturation was not addressed such as to Grounded theory, instead the number of critical incidents were determined by the study purpose, i.e. a purposeful sampling, rather than the quantity of participants, in accordance with Flanagan (1954). The literature suggests, for more straightforward tasks or roles, 50 to 100 incidents to be adequate (Flanagan, 1954; Fridlund et al, 2017). In this study, participants reported a total of 99 critical incidents, which yielded 491 experiences and actions in total. This is added in discussion of methods.<br><br>The nine-question interview guide for the CIT was used according to the well-proven Fridlund

	et al (2017) procedure. That study was to elucidate the development and current status of the CIT with focus on its fundamental definitions, guidelines, and pros and cons when applied in nursing and healthcare sciences.<br><br>The main questions were adapted for this study and will, as you suggest, be added to the table. See table 2.
**Data Analysis**	
How was analysis done? Was it by hand or using a qualitative analysis software?<br><br>How many researchers completed the coding? How was inter-rater reliability determined? (Again, important to demonstrate reliability of<br><br>qualitative methods.)	Thank you for the valuable input. This is added in the text:<br><br>The analysis was done manually by the authors. The first author read the interview transcripts multiple times, extracting and condensing experiences and actions, and assigning specific codes manually. The aggregation of codes into sub-categories, categories, and main areas was also done through a manual, systematic sorting and individual analysis process within the author group.<br><br>All researchers were involved in the analysis process. It was a collaborative effort to scrutinizing and systematizing the data to validate the results at each step. All researchers reached a consensus on the categories and areas that emerged, ensuring that the analysis was well-supported by the data. When a challenge or a diverse view arose, the researchers revisited the transcribed interview to gain a comprehensive understanding and choose the most reasonable codes that best matched the data. Inter-rater reliability was not specifically addressed.
Tables 3 & 4: Suggest moving it to FINDINGS sections, after first short paragraph.	Thank you so much for this accurate input, these tables are now under findings.
**Findings**	
This is more so regarding Tables 3 & 4. First, I'd suggest<br><br>reorganizing the columns in the table. "Main Area" on the left; also recommend calling	Thank you so much for this accurate input. The columns in Tables 3 and 4 have been reorganized. Main Area on the left. Then Category, and sub-category on right. However, the main area is not the same as a theme in a latent content analysis. The name "main area"

this a “Main theme.” Then Category, and sub-category on right.<br><br>I would also recommend emphasizing the raw data (direct quotes) more. Within the text, they don’t really stand out. Consider making them a separate line, or making a 4th column to the right in the table with the example quotes.	indicates the descriptive manifest level of the analysis.<br><br>Thank you for the valuable comments. The quotes are on a separate line in the text now.
**Discussion of findings**	
Don’t just repeat the results.<br><br>This section needs more depth. You mention how the findings “highlight” this and that,<br><br>but how? Be specific. What is the “so, what?” of the findings.	Thank you for the valuable comments. The discussion sections are further developed. Changes are highlighted in yellow in the manuscript.
**Discussion of methods**	
The very first sentence (lines 51-52) left me<br><br>hanging. Suggest adding paragraph (page 10, lines 14-30) immediately following here.<br><br>Then address each one of those constructs, in order, with the specifics. Page 10, line<br><br>21: should be “the majority of PTFs WERE men.”	This section is rewritten in according to reviewer’s comments.
**Conclusions and implications**	
The first paragraph is just repeat of the<br><br>results again. Instead, synthesize the review of literature and study results collectively.<br><br>The next sections (page 10, line 44, to page 11, line 6) is what I would have liked to see<br><br>in the Discussion section. Consider putting it there and expanding on the<br><br>recommendations based on the evidence and previous literature.	The conclusion is rewritten as follows: This study underscores the profound impact on the daily lives, emotional well-being, and family dynamics of part-time firefighters' families. Family members balance their personal inconveniences with the community's safety needs, navigating the conflict between their own needs and the demands of the firefighter's role. Despite these challenges, they strive to provide emotional support and maintain open communication to address mental health aspects. The decision to become a PTF is a collective family commitment, fostering mutual support and shared responsibility.<br><br>Thank you for your comments. That sections are incorporated in the discussion now.

**COREQ Checklist**	
COREQ Checklist does not appear correct. Unable to find Item #1 on page 11.<br><br>Item #2: Credentials not listed on title page. This is PhD, MD, etc.<br><br>Item #5: cannot locate training and experience. This could be added where describing who conducted the<br><br>interview and analyzed the data, if needed. #10: Sampling method not identified.<br><br>#13:<br><br>not addressed.<br><br>#17 and #19: not addressed.<br><br>Also see my comments above regarding<br><br>data collection. #20: Why are field notes during interviews "N/A"? Field notes are<br><br>important in qualitative studies.<br><br>#22: Saturation not addressed.<br><br>#24: not addressed	Thank you for noticing this. The checklist as well as the main document is clarified:<br><br>#1 is now on page 3<br><br>#2 Credentials are now listed on title page<br><br>#5 is now on page 4 (and title page)<br><br>#10 clarified on page 3<br><br>#13 clarified page 3<br><br>#17 clarified page 3<br><br>#19 clarified page 3<br><br>#20 The first author took field notes during interviews, but the analysis process was conducted from the transcribed audio file.<br><br>#22 Saturation was not addressed such as to Grounded theory, instead the number of critical incidents were determined by the study purpose, i.e. a purposeful sampling, rather than the quantity of participants, in accordance with Flanagan (1954).<br><br>#24 Clarified page 4

VERSION 2 - REVIEW

**REVIEWER NAME**	Sharp, Marie-Louise
**REVIEWER AFFILIATION**	King's College London, Psychological Medicine
**REVIEWER CONFLICT OF INTEREST**	None
**DATE REVIEW RETURNED**	23-Aug-2024

**GENERAL COMMENTS**	Thank you for your revised manuscript. I think the additions you have incorporated from the reviews strengthen the work. This is a neat piece of work that gives more qualitative evidence on part-time firefighters. There are some minor issues I have identified in the discussion that should be addressed.<br><br>Pg11 - reference to a smart phone app seems to be dropped in with no context and no citations - so I am unclear where this suggestion comes from?

	Pg12 - has a huge paragraph - can this be broken up?<br>Pg 12 - line 37/38 -'Achieving and work life balance...etc' this needs a citation.

**REVIEWER NAME**	Jones, Sara
**REVIEWER AFFILIATION**	University of Arkansas for Medical Sciences, College of Nursing
**REVIEWER CONFLICT OF INTEREST**	None
**DATE REVIEW RETURNED**	05-Aug-2024

**GENERAL COMMENTS**	All of my comments were adequately and excellently addressed. I have no additional comments. This final work contributes to scientific knowledge.

VERSION 2 – AUTHOR RESPONSE

**Reviewer 1**	
Pg11 - reference to a smart phone app seems to be dropped in with no context and no citations - so I am unclear where this suggestion comes from?	Thank you for this valuable comment. This is rewritten and added in the discussion:<br><br>To address some of this frustration and to alleviate the family member´s need for planning, a smart phone application with a time prognosis is valuable. Several Swedish fire and rescue services utilize an application called *Respons*, designed to enhance communication and information management for emergency services [25]. Granting family members limited access to this tool could be beneficial, as it would aid in planning and reduce uncertainty through improved forecasting.
Pg12 - has a huge paragraph - can this be broken up?	Thank you for noticing this. The paragraph is now broken up into smaller parts.
Pg 12 - line 37/38 -'Achieving and work life balance...etc' this needs a citation.	Tank you! This sentence is reformulated, and a reference is added:<br><br>Maintaining a healthy work-family balance is crucial for the long-term retention of firefighters, as it reduces the strain on their personal lives and encourages continued participation [3].
